# Impact of the body mass index on the retention of the anorectal mucosa after double-stapled ileal pouch-anal anastomosis for ulcerative colitis

**DOI:** 10.1186/s12876-023-02667-w

**Published:** 2023-02-08

**Authors:** Hideaki Kimura, Kenichiro Toritani, Reiko Kunisaki, Kenji Tatsumi, Kazutaka Koganei, Akira Sugita, Itaru Endo

**Affiliations:** 1 Inflammatory Bowel Disease Center, Yokohama City University Medical Center, 4-57, Urafune-Cho, Minami-Ku, Yokohama, Kanagawa 232-0024 Japan; 2 Inflammatory Bowel Disease Center, Yokohama Municipal Citizen’s Hospital, Yokohama, Japan; 3 Gastroenterological Surgery, Yokohama City University, Yokohama, Japan

**Keywords:** Body mass index, Double-stapled ileal pouch-anal anastomosis, Length of the retained mucosa, Retention of the anorectal mucosa, Ulcerative colitis

## Abstract

**Background:**

Double-stapled ileal pouch-anal anastomosis (DS-IPAA) is easy to construct and has a good functional outcome in patients with ulcerative colitis (UC). However, retention of the anorectal mucosa may lead to a subsequent risk of inflammation and neoplasia. This study aimed to identify factors associated with the retention of a large amount of anorectal mucosa after DS-IPAA.

**Methods:**

The medical records of 163 patients who had undergone one-stage total proctocolectomy and DS-IPAA for UC between 2007 and 2020 were retrospectively reviewed. The patients were divided into two groups according to the length of the retained mucosa. The high anastomosis group was defined as having a retained mucosal length of ≥ 30 mm in the anterior or posterior wall. Clinical factors were compared between the high and low anastomosis groups.

**Results:**

The high anastomosis group showed a significantly higher body mass index (BMI) (high vs. low: 23.2 vs. 19.0), longer operation time (304 vs. 263) and greater blood loss (357 vs. 240). In the multivariate analysis, high BMI was the only factor significantly associated with high anastomosis (odds ratio 1.32). There was a positive correlation between BMI and the length of the retained mucosa.

**Conclusions:**

In DS-IPAA, BMI showed the strongest association with the retention of a large amount of the anorectal mucosa. In high BMI patients, although the risk of inability of anastomosis is little than that of IPAA with mucosectomy, the possible retention of a large amount of mucosa should be considered.

## Background

Both stapled and hand sewn ileal pouch-anal anastomosis (IPAA) are the standard procedures after restorative proctocolectomy in patients with ulcerative colitis (UC). Stapled IPAA is usually performed with a double stapling technique without mucosectomy (DS-IPAA), whereas hand sewn IPAA is made on the dentate line with mucosectomy [1]. The advantage of stapled IPAA is that it is easy to construct and has a good functional outcome [2–6].

Generally, stapled IPAA is made in the surgical anal canal with preservation of the upper anal canal mucosa, including the anal transitional zone, which is one of the reasons for the good postoperative anal function in comparison to hand sewn IPAA [7]. On the other hand, preservation of the anorectal mucosa may lead to a subsequent risk of developing inflammation and cancer. Therefore, the length of the retained anorectal mucosa should be minimal, even in the case of stapled anastomosis without mucosectomy. The ECCO statement shows that the maximum length of anorectal mucosa between the dentate line and anastomosis should not exceed 2 cm [3].

A new double stapling technique, partially intra-anal canal anastomosis, was also developed to reduce residual mucosa [8]. Nevertheless, DS-IPAA sometimes leads to the retention of a large amount of rectal mucosa, and the specific factors that prevent appropriate anastomosis are not well known. The aim of the present study was to identify factors associated with the retention of a large amount of anorectal mucosa after DS-IPAA.

## Methods

### Patients

**Fig. 1:**
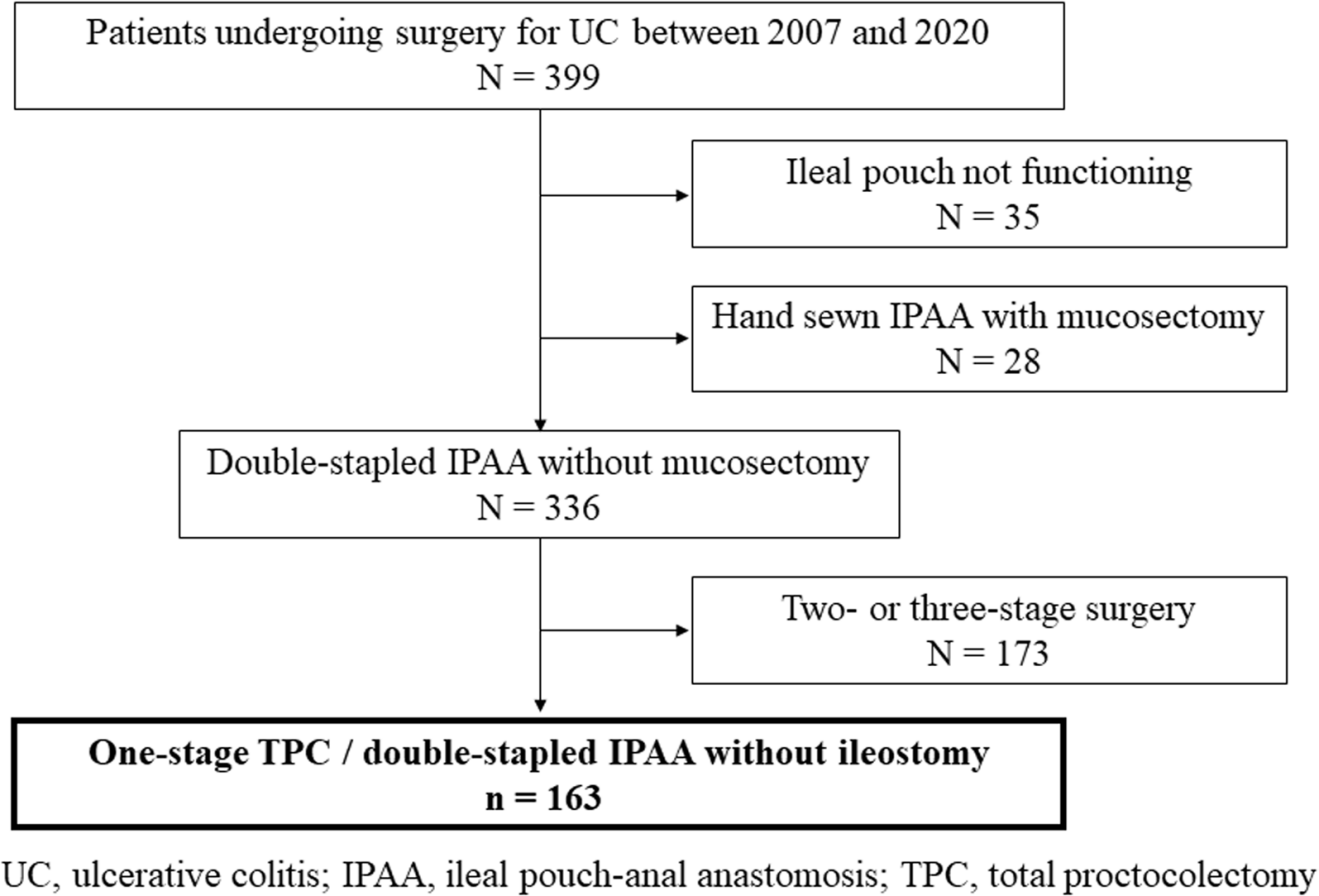
Primary surgery for UC in our institution. UC, ulcerative colitis; IPAA, ileal pouch-anal anastomosis; TPC, total proctocolectomy

This was a retrospective, exploratory cross-sectional study. A total of 399 patients underwent surgery for UC in Yokohama City University Medical Center between January 2007 and December 2020. Among these, the medical records of 163 patients who underwent one-stage total proctocolectomy and DS-IPAA were retrospectively reviewed. Because there were marked differences in preoperative conditions between 1-stage and 2- or 3-stage surgery, only 1-stage surgery was targeted in this study (Fig. 1). DS-IPAA was mainly indicated for refractory and severe cases, and one-stage surgery was mainly performed for refractory cases. In contrast, cases of colorectal cancer and severe stenosis or fistula in the anorectal area were not indicated for DS-IPAA; in these cases, hand-sewn IPAA was performed.

### Surgical procedure

**Fig. 2:**
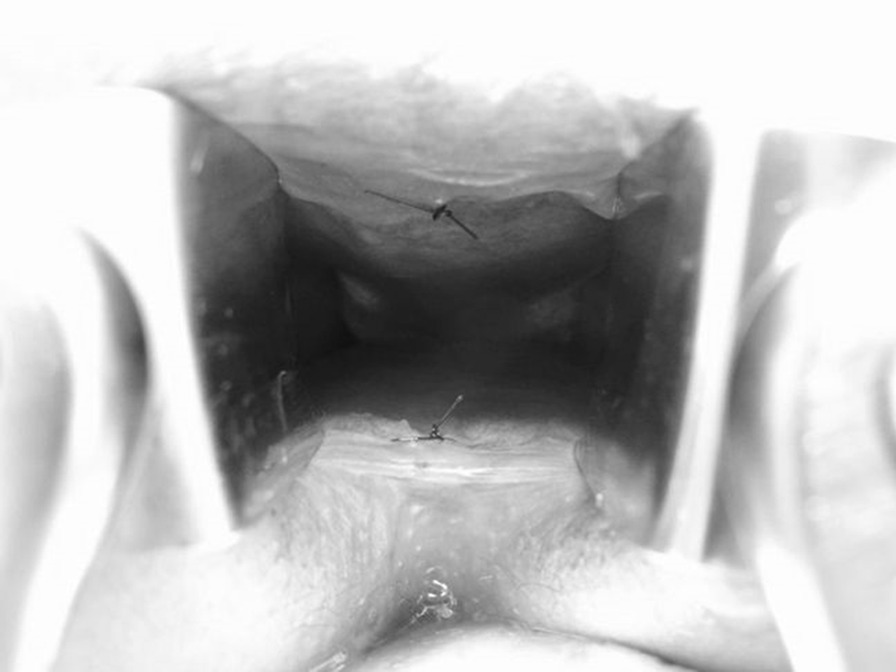
Thread marking of the dentate line. Thread marking of the anterior and posterior walls of the dentate line is performed before the operation

**Fig. 3:**
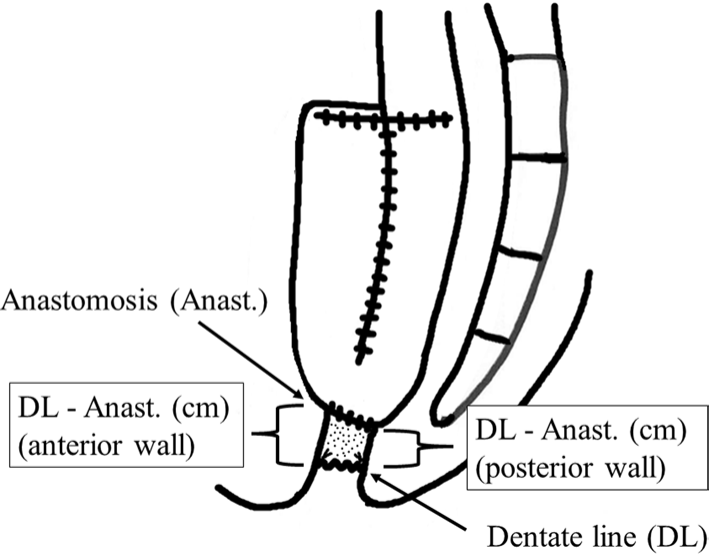
Definition of the retained rectal mucosa. The anterior and posterior wall of the length of the retained rectal mucosa (from the dentate line to the site of anastomosis) were measured immediately after the anastomotic procedure. DL, dentate line; Anast., anastomosis

Thread marking of the anterior and posterior walls of the dentate line was performed before the operation in order to confirm the site of resection and anastomosis during the operation (marking technique) (Fig. 2). Colectomy and rectal resection were performed with hand-assisted laparoscopic surgery (HALS) or conventional open surgery. The surgeon ensures that the apex of the proposed ileal pouch reaches 2 cm below the inferior margin of the pubis before rectal resection. Rectal resection is performed in the TME layer, and the rectococcygeal muscle (so-called hiatal ligament) is then carefully resected. The anorectal junction is sutured and divided using a suture device for laparotomy, which is narrower than that for laparoscopic surgery and which can be horizontally inserted deeper into the pelvis. An anal dilator is used to dilate the anus before anastomosis is performed. In all cases, anastomosis was performed with conventional double stapling technique with a 31-mm circular stapler. The anterior and posterior wall of the length of retained anorectal mucosa (from the dentate line to the site of anastomosis) were measured immediately after the anastomotic procedure (Fig. 3). An anal drain was inserted in the ileal pouch and removed at 7–10 days after surgery. All surgical operations in this study were performed by one lead surgeon (HK).

### Methods

The patients were divided into two groups according to the length of the retained rectal mucosa. The high anastomosis group was defined as having a retained mucosal length of ≥ 30 mm in the anterior or posterior wall, whereas the low anastomosis group was defined as having a retained mucosal length of < 30 mm in the anterior and posterior wall. The high anastomosis group was defined as ≥ 30 mm to emphasize the notion that the retained mucosal length in the high anastomosis group is much longer than the average. Clinical factors were compared between the two groups, and related factors were identified. The correlation between the extracted factors and the length of retained anorectal mucosa was also analyzed.

Inflammation of the retained anorectal mucosa was assessed by the Mayo endoscopic score. Cuffitis was defined as the presence of endoscopic and histologic inflammation of the rectal cuff [9], and pouchitis was defined as a modified PDAI index score of ≥ 5 [10]. Incontinence was described as spotting (staining of the undergarments not exceeding 3 cm in diameter) or soiling (staining more than 3 cm in diameter).

### Outcomes

The primary outcome was to determine the related factors associated with the retention of a large amount of anorectal mucosa. The correlation between the extracted factors and the length of the retained anorectal mucosa and postoperative outcomes were also examined.

### Statistical analysis

For the statistical analysis, comparisons between different groups were performed using non-parametric methods. A univariate analysis was used to identify factors associated with the retention of a large amount of anorectal mucosa and to compare postoperative outcomes. Categorical variables were compared using a X^2^ test or Fisher's exact test. Continuous variables were expressed as the median or average and range and were compared using the Mann–Whitney U test. All variables (except the length of the retained anorectal mucosa) that showed a *p* value of < 0.05 were entered into a multiple logistic regression analysis. Odds ratios (ORs) and 95% confidence intervals (CIs) were calculated. Pearson's correlation analysis was used to determine the correlation between the extracted factors and the length of the retained rectal mucosa. *p* values of < 0.05 were considered to indicate statistical significance.

## Results

### Patient characteristics

**Fig. 4:**
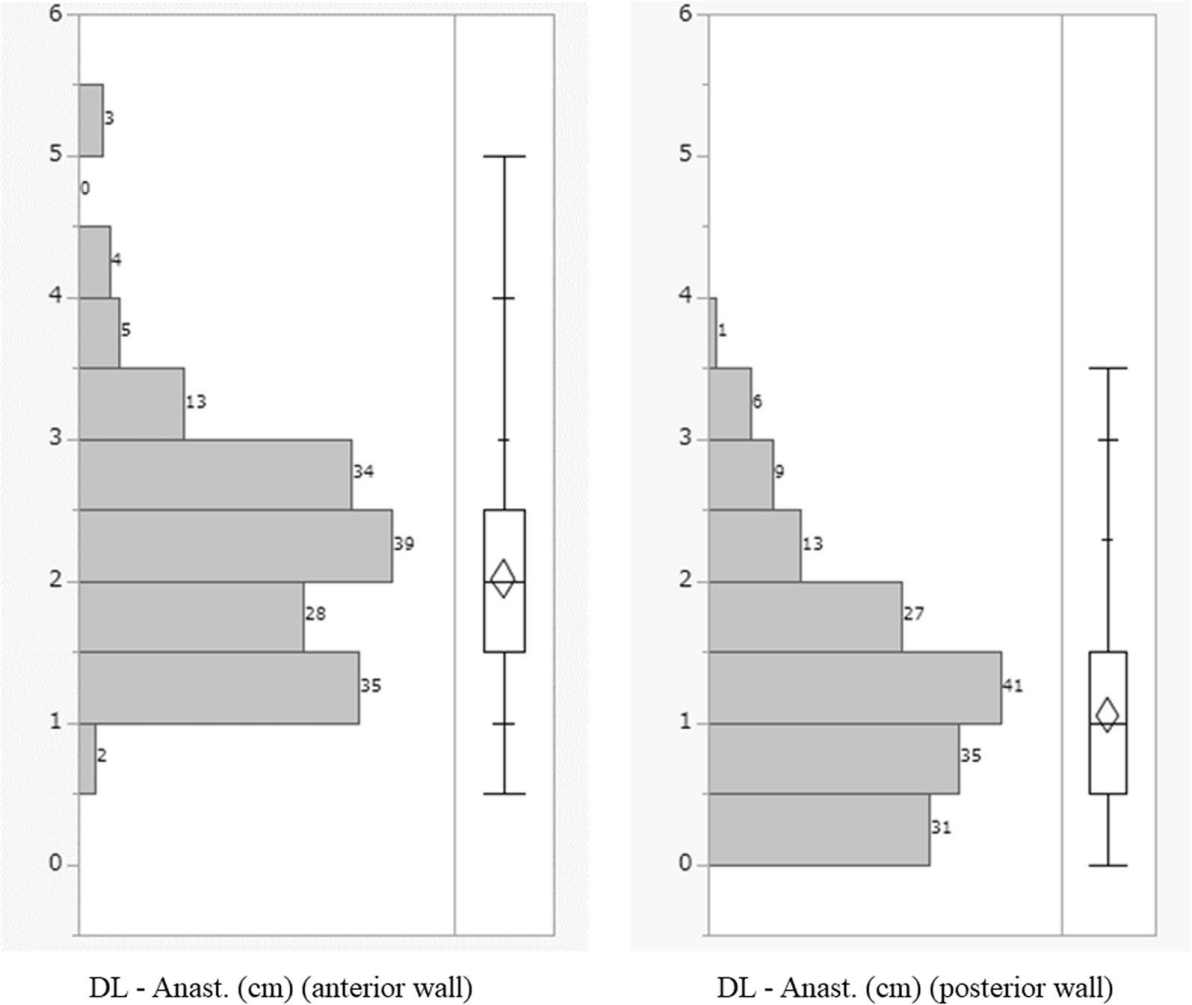
The length of the retained rectal mucosa

The patient characteristics are shown in Table 1. The study population included 68 women and 95 men (median age at surgery: 37 years). The most common indication for surgery was intractability (149/163 patients), because only a one-stage surgery was targeted. Surgical procedures included HALS (n = 146) and conventional open surgery (n = 17). The average length of the retained anorectal mucosa (from the dentate line to the site of anastomosis) was 2.0 cm on the anterior wall and 1.1 cm on the posterior wall (Fig. 4). There were no cases with intraoperative abandonment of anastomosis due to inability for the pouch to reach the anal side in either of the groups.

**Table 1:** Characteristics of patients and the univariate analysis

	Total	High anastomosis	Low anastomosis	*p* value
	(n = 163)	(n = 25)	(n = 138)	
Sex (male:female)	95:68	19:6	76:62	0.051
Age at surgery, median year (range)	37 (10–82)	43 (14–72)	36 (10–82)	0.254
Disease duration, median month (range)	60 (1–444)	60 (9–302)	63 (1–444)	0.967
BMI, median (range)	19.3 (11.3–37.1)	23.2 (18.7–37.1)	19.0 (11.3–30.1)	< 0.0001*
Type of colitis (total:left)	153:10	24:1	129:9	0.629
Surgical indication, cases (%)				
Severe	5 (3)	0 (0)	5 (4)	
Intractability	149 (91)	24 (96)	125 (90)	
Cancer/dysplasia	9 (6)	1 (4)	8 (6)	0.578
Preoperative medical therapy				
Total dose of prednisolone, mg (range)	4480 (0–80,000)	6000 (0–40,000)	4100 (0–80,000)	0.415
Use of biologics, cases (%)	76 (47)	10 (40)	66 (48)	0.471
Use of immunomodulators, cases (%)	86 (53)	14 (56)	72 (52)	0.724
Emergency surgery, cases (%)	2 (1)	0 (0)	2 (1)	1.000
ASA physical status classification system, cases (%)				
I	12 (7)	3 (12)	9 (7)	
II	145 (89)	22 (88)	123 (89)	
II E	2 (1)	0 (0)	2 (1)	
III	4 (3)	0 (0)	4 (3)	
IV, V	0 (0)	0 (0)	0 (0)	0.582
Surgical procedure, cases (%)				
Laparoscope assisted surgery (HALS)	146 (90)	25 (100)	121 (88)	
Conventional open surgery	17 (10)	0 (0)	17 (12)	0.077
Operation time, median minutes (range)	267 (175–426)	304 (188–426)	263 (175–394)	0.001*
Blood loss, median grams (range)	250 (0–1500)	357 (143–1315)	240 (0–1500)	0.004*
Intraoperative transfusion, cases (%)	9 (6)	2 (8)	7 (5)	0.555
Length of the retained anorectal mucosa, average cm (range)				
Anterior wall	2.0 (0.5–5.0)	3.5 (3.0–5.0)	1.7 (0.5–2.5)	< 0.0001*
Posterior wall	1.1 (0–3.5)	2.1 (0.5–3.5)	0.9 (0–2.5)	< 0.0001*
Intraoperative abandonment of anastomosis due to inability for the pouch to reach the anal side, cases (%)	0 (0)	0 (0)	0 (0)	NA

BMI, body mass index; ASA, American Society of Anesthesiologists; HALS, hand-assisted laparoscopic surgery

*< 0.05

### Factors associated with the retention of a large amount of the anorectal mucosa

The univariate analysis showed that body mass index (BMI) (23.2 vs. 19.0; *p* < 0.0001), operation time (304 vs. 263; *p* = 0.001) and blood loss (357 vs. 240; *p* = 0.004) were significantly higher in the high anastomosis group (Table 1). There were no significant differences in sex, age at surgery, disease duration, type of colitis, surgical indication, preoperative medical therapy, emergency surgery, American Society of Anesthesiologists (ASA) physical status, or surgical procedure (laparoscopic assisted surgery or open surgery). The multivariate logistic regression analysis showed that BMI was the only factor that was significantly associated with high anastomosis (odds ratio 1.32, *p* < 0.0001) (Table 2).

**Table 2:** Multivariate logistic regression analysis

	Odds ratio	95% CI	*p* value
Body mass index	1.32	1.16–1.50	< 0.0001*
Operation time (minutes)	1.01	1.00–1.02	0.342
Blood loss (grams)	1.00	1.00–1.00	0.579

*< 0.05

### The correlation between the extracted factors and the length of the retained anorectal mucosa

**Fig. 5:**
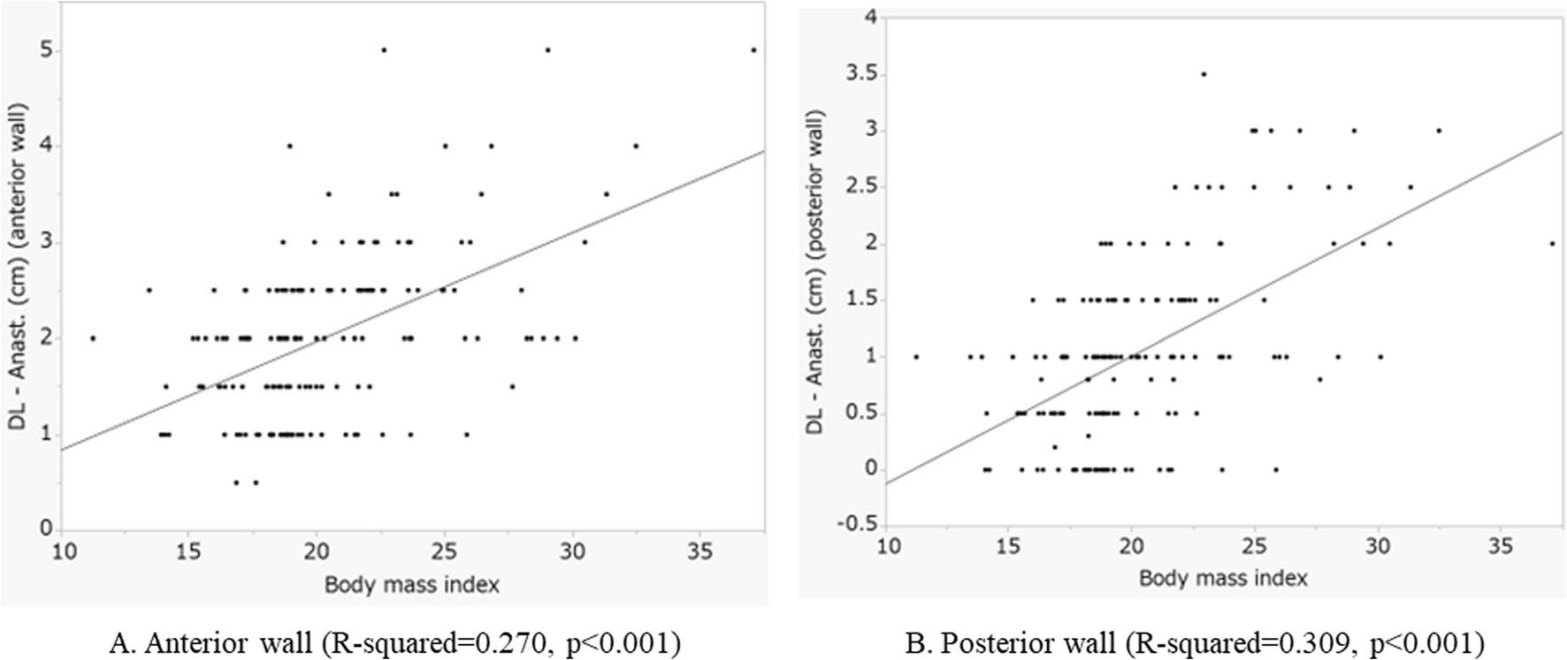
Correlation between the length of the retained rectal mucosa (from the site of anastomosis to the dentate line) and body mass index. Anast, Anastomosis; DL, Dentate line. **A** Anterior wall (Anast—DL (cm) (anterior wall) = − 0.334227 + 0.1152773*Body mass index, R-squared = 0.266, *p* < 0.001). **B** Posterior wall (Anast—DL (cm) (posterior wall) = − 1.210927 + 0.1100184*Body mass index, R-squared = 0.285, *p* < 0.001)

The correlation between BMI and the length of the retained anorectal mucosa was examined. There was positive correlation between BMI and the length of the retained anorectal mucosa in both the anterior and posterior wall (anterior wall: R-squared = 0.270, *p* < 0.001, posterior wall: R-squared = 0.309, *p* < 0.001) (Fig. 5).

### Postoperative outcomes

There were no differences in the bowel function at 1 year after surgery (bowel movements per day, nocturnal defecation, soiling, spotting, difficulty in distinguishing feces from flatus, usage of antidiarrheal agents) between the high and low anastomosis groups. There were also no differences in the incidence of inflammation, cuffitis, pouchitis, or neoplasia of the retained mucosa between two groups (median observational period: 62 months) (Table 3).

**Table 3:** Postoperative outcomes according to the retained anorectal mucosa

	Total	High anastomosis	Low anastomosis	*p* value
	(n = 163)	(n = 25)	(n = 138)	
Bowel function at 1 year after surgery				
Bowel movement per day, average times (range)	7.9 (3–20)	8.5 (4–20)	7.8 (3–16)	0.398
Nocturnal defecation, average times (range)	0.8 (0–7)	1.1 (0–5)	0.8 (0–7)	0.075
Soiling, cases (%)	15 (9)	0 (0)	15 (11)	0.095
Spotting, cases (%)	56 (34)	9 (36)	47 (34)	0.650
Difficulty in distinguishing feces from flatus, cases (%)	49 (30)	4 (16)	45 (33)	0.112
Usage of antidiarrhal agents	113 (69)	19 (76)	94 (68)	0.212
Outcome of the retained anorectal mucosa				
(median observational period was 62 month)				
Inflammation, cases (%)				
Mayo 0	32 (21)	2 (10)	30 (23)	
Mayo 1	76 (51)	14 (67)	62 (48)	
Mayo 2	25 (17)	4 (19)	21 (16)	
Mayo 3	16 (11)	1 (4)	15 (13)	0.297
NA	14	4	10	
Cuffitis, cases (%)	41 (25)	5 (24)	36 (28)	0.682
Cuffitis with symptoms, cases (%)	11 (7)	1 (5)	10 (8)	0.620
Cuffitis without pouchitis, cases (%)	4 (2)	0 (0)	4 (3)	0.412
Pouchitis, cases (%)	22 (13)	2 (8)	20 (14)	0.382
Dysplasia or cancer development, cases (%)	0 (0)	0 (0)	0 (0)	NA

NA, not available

*< 0.05

## Discussion

Our study showed that BMI showed the strongest association with the retention of a large amount of anorectal mucosa in DS-IPAA. And there was positive correlation between BMI and the length of retained mucosa. In DS-IPAA, the length of retained mucosa is defined by the site of anastomosis, which is defined by the suturing and dividing site of the anorectal region. To reduce length of the retained mucosa, we have been using a marking technique and adequate dissection of the hiatal ligament. As a result, the average length of the retained mucosa (from the dentate line to the anastomosis) was 2.0 cm in the anterior wall and 1.1 cm in the posterior wall in this study. On the other hand, in some patients with high BMI values, up to 5.0 cm of retained mucosa remained. In high BMI patients, the main reason for the retention of a large amount of the anorectal mucosa in DS-IPAA is difficulty in stapling and dividing the rectum at the appropriate site in the deep pelvic region. The suture device does not go deep enough, and the site of stapling and division is far away from the dentate line, resulting in an increase in retained mucosa. Difficulties in deep pelvic manipulation led to various postoperative complications in surgery for UC [11–13]. Increased blood loss and a prolonged operative time are observed in patients with high BMI values [2, 13]. According to the univariate analysis in this study, the operation time was longer, and the blood loss was higher in the high anastomosis group. The high anastomosis group included many patients with high BMI values, so these results seemed to suggest of the difficulty of intrapelvic manipulation in patients with high BMI values. In the present study, there was a trend toward more men being included in the high anastomosis group (*p* = 0.051), but no significant sex difference was detected. In fact, surgery is often more difficult in men than in women due to the former’s small pelvis. However, while surgery may be more difficult in men, our data suggested that the BMI of individual cases may have had a greater influence on the retained mucosal length than sex differences.

In UC surgery, high BMI has been reported to be a risk factor for intraoperative abandonment of IPAA due to inability of the ileal pouch to reach the anus [14–16]. Thickened and unstretched ileal mesentery due to fat deposition is thought to be the main cause [15]. In patients undergoing DS-IPAA the risk of being unable to complete anastomosis is relatively low in comparison to hand sewn IPAA with mucosectomy because the anastomotic site is cephalad. In this study, there were no cases in which anastomosis was abandoned during surgery due to the inability of the pouch to reach the anal side. The amount of retained mucosa was increased in high BMI patients, which may have made it easier for the pouch to reach the anal side. In any event, one of the advantages of DS-IPAA is that the anastomotic position can be adjusted. If mucosectomy from the dentate line is performed first, anastomosis will be impossible if the ileal pouch cannot be reached, resulting in permanent ileostomy. Even with DS-IPAA, it is possible that the ileal pouch will not be able to be reached later if the anorectal side is divided first. To prevent this, whether the ileal pouch reaches the anal side must be sufficiently confirmed before the anorectal side is removed (pouch reach test). The pouch reach test checks whether the apex of the ileal pouch extends beyond the inferior margin of the pubis (it is recommended that it exceeds 3–4 cm) [16]. If the pouch does not sufficiently reach even with ligation of blood vessels and adequate extension of the ileal mesentery, one option is to avoid mucosectomy and convert to stapled IPAA. Horio et al. reported that 2.4% of patients scheduled for mucosectomy required conversion to staple anastomosis due to insufficient pouch reach [11]. In contrast, in cases with colitis-associated cancer, mucosectomy is strongly recommended because the risk of tumor development in the retained mucosa is considered higher [17]. In such cases, informed consent should be obtained preoperatively to determine whether conversion to DS-IPAA or abdominoperineal resection should be performed if the pouch cannot reach the anal side.

To reduce the length of the retained mucosa in patients with a high BMI, preoperative weight loss is recommended. If the surgery is a planned procedure, it is preferable for the patient to lose as much weight as possible before the operation. If the patient has a high BMI at the time of initial surgery, it may be effective to perform a staged surgery without reconstruction (subtotal colectomy and ileostomy), and to perform reconstructive surgery after weight loss [2, 15]. A technological solution would be ideal, but a quick solution is difficult to achieve. Advances in surgical devices are expected.

There was no difference in the postoperative bowel function between the high and low anastomosis groups. If the anal transitional zone is preserved, the result seems to be that leaving a few centimeters more mucosae does not improve bowel function, and it is no need to increase the amount of retained anorectal mucosa in order to maintain bowel function in DS-IPAA. In terms of the long-term prognosis, there were no differences in the incidence of inflammation, dysplasia, or cancer of the retained mucosa between the two groups during the 62-month observation period. The severity of inflammation of the retained mucosa did not correlate with the length of the retained mucosa in our results. Kayal et al. reported that a rectal cuff length > 20 mm was a possible risk factor for cuffitis [18, 19]. However, it was not clear how much mucosa remained in the > 20-mm group. In the present study, the high anastomosis group had a mean anterior wall of 3.5 cm and posterior wall of 2.1 cm, while the low anastomosis group had an anterior wall of 1.7 cm and posterior wall of 0.9 cm, suggesting that the difference in mucosal length between the two groups was not that great. Alternatively, the fact that inflammation of the cuff was seen in 28% of cases even in the low anastomosis group may indicate that, within a certain range of retained mucosal length, the severity of inflammation of the cuff is determined by the disease activity in individual cases, not by the mucosal length [20]. There is no doubt that the risk of tumor development increases with a longer retained mucosa. Stapled IPAA is reported to be eight times riskier than hand sewn IPAA plus mucosectomy [17], and ileorectal anastomosis is reported to carry an even greater risk [21]. These reports indicate that the retained mucosal length correlates with the risk of tumor development. The short observation period and small number of cases in the present study are considered inadequate for evaluating tumor development. Further studies of a larger number of cases with a longer follow-up are needed.

The present study included some limitations with regard to the surgical procedure and patient selection. First, this study was a retrospective chart review, and the length of the retained mucosa was determined by the best effort in each surgery. In most cases, the length of residual mucosa was as small as possible; however, factors other than technical factors may need to be considered. Second, this was a single institutional study. The generalizability of single institutional study is usually considered to be lower in comparison to multicenter studies. However, this study was associated with some advantages. Detailed information was obtained, and all cases were free from missing data, including the length of retained anorectal mucosa. Furthermore, surgeon-related technical bias could be reduced because all surgeries in this study were performed by one lead surgeon. The third limitation was racial bias. In this study, almost all patients were Asian (Japanese). The mean BMI of Japanese people is 23.68, whereas that of the people in the United States is 29.01 and that of the people in Europe (e.g., the United Kingdom) is 27.48 [22]. Thus, the results in very high BMI patients were not investigated in this study. However, few studies have shown the detailed relationship between BMI and the length of the retained anorectal mucosa. We therefore believe that the results of this study will provide useful information for patients of any race and from any country.

## Conclusions

In DS-IPAA, BMI was the factor most strongly associated with the retention of a large amount of anorectal mucosa and there was positive correlation between BMI and the length of the retained mucosa. In high BMI patients, although the risk of permanent ileostomy due to failure to reach the ileal pouch to the anal side is lower in comparison to IPAA with mucosectomy, the risk of the retention of a large amount of the anorectal mucosa should be considered. Weight loss and staged surgery should be recommended in such patients.

## Data Availability

The datasets used and analyzed during the current study are available from the corresponding author on reasonable request.

## Abbreviations

DS-IPAA: Double-stapled ileal pouch-anal anastomosis
UC: Ulcerative colitis
BMI: Body mass index
IPAA: Ileal pouch-anal anastomosis
HALS: Hand-assisted laparoscopic surgery
TME: Total mesorectal excision
PDAI: Pouchitis disease activity index
ASA: American Society of Anesthesiologists
